# Geographically-weighted regression of knowledge and behaviour determinants to anti-malarial recommending and dispensing practice among medicine retailers in western Kenya: capacitating targeted interventions

**DOI:** 10.1186/s12936-016-1599-5

**Published:** 2016-11-21

**Authors:** Andria Rusk, Linda Highfield, J. Michael Wilkerson, Melissa Harrell, Andrew Obala, Benjamin Amick

**Affiliations:** 1 Dominican University of California, San Rafael, CA USA; 2 The University of Texas School of Public Health, Pressler Dr, Houston, TX USA; 3 Moi University School of Medicine, Nandi Rd, Eldoret, Kenya; 4 Webuye Demographic Surveillance Site Scientific Steering Committee, Eldoret, Kenya; 5 Department of Health Policy and Management, Robert Stempel College of Public Health & Social Work, Florida International University, Miami, FL USA; 6 Institute for Work & Health, Toronto, Canada

**Keywords:** Behavior, Private sector, Geographically weighted regression, Malaria, Antimalarial drugs

## Abstract

**Background:**

Most patients with malaria seek treatment first in retail drug shops. Myriad studies have examined retailer behaviours and characteristics to understand the determinants to these behaviours. Geospatial methods are helpful in discovering if geographic location plays a role in the relationship between determinants and outcomes. This study aimed to discover if spatial autocorrelation exists in the relationship between determinants and retailer behaviours, and to provide specific geographic locations and target behaviours for tailoring future interventions.

**Methods:**

Retailer behaviours and characteristics captured from a survey deployed to medicine retailers in the Webuye Demographic and Health Surveillance Site were analysed using geographic weighted regression to create prediction models for three separate outcomes: recommending the first-line anti-malarial therapy to adults, recommending the first-line anti-malarial therapy to children, and selling that therapy more than other anti-malarials. The estimated regression coefficients for each determinant, as well as the pseudo R^2^ values for each final model, were then mapped to assess spatial variability and local areas of best model fit.

**Results:**

The relationships explored were found to be non-stationary, indicating that spatial heterogeneity exist in the data. The association between having a pharmacy-related health training and recommending the first-line anti-malarial treatment to adults was strongest around the peri-urban centre: comparing those with training in pharmacy to those without training (OR = 5.75, *p* = 0.021). The association between knowing the first-line anti-malarial and recommending it to children was strongest in the north of the study area compared to those who did not know the MOH-recommended anti-malarial (OR = 2.34, *p* = 0.070). This is also the area with the strongest association between attending a malaria workshop and selling the MOH-recommended anti-malarial more than other anti-malarials, compared to retailers who did not attend a workshop (OR = 2.38, *p* = 0.055).

**Conclusion:**

Evidence suggests that spatial heterogeneity exists in these data, indicating that the relationship between determinants and behaviours varies across space. This is valuable information for intervention design, allowing efforts to focus on those factors that have the strongest relationship with their targeted behaviour within that geographic space, increasing programme efficiency and cost-effectiveness.

## Background

Malaria is one of the world’s most widespread parasitic diseases, causing an estimated 214 million cases of disease, and approximately 438,000 deaths in 2015 [1]. The incidence rate in Kenya was 8200 cases per 100,000 population in 2012, with particular burden among children under the age of 5 years [2]. In Kenya, several studies have shown that families first seek treatment for malaria from retail drug shops rather than from the public health sector [3–5]. However, assessments of private medicine retailer practice have shown that retailers often recommend, prescribe, and dispense outdated or inappropriate treatments for malaria [6–9]. The Kenya Ministry of Health recommendation for first-line treatment of uncomplicated malaria changed from sulfadoxine-pyrimethamine (SP), also known by the brand name Fansidar**®**, to artemisinin-based combination therapies therapy (ACT) in 2006. Since that time, the importance of exploring determinants to medicine retailer dispensing behaviours has grown, to ensure providers are adhering to the new policies and customers are getting access to the more effective treatment [10].

The change in treatment recommendation, combined with treatment-seeking behaviour in retail drug shops, spurred the launch of the Affordable Medicines Facility—Malaria (AMFm) programme, which sought to subsidize the cost of ACT in the retail sector to encourage their sale over other anti-malarials. This effort moved medicine retailers even closer to the forefront of malaria treatment and control efforts. As a result, understanding the determinants of medicine retailer behaviour, and discovering ways to improve their knowledge and practice, has become the focus of numerous training and education programmes [11–13].

However, interventions are often not as effective as they could be. Improvements are frequently inconsistent across study sites, showing behaviour improvements in one area and not in other areas. For example, a Ministry of Health (MoH) intervention assessment by Abuya and colleagues used a cluster randomized controlled trial to compare intervention efficacy across three districts in Kenya [6]. The interventions examined focus on improving anti-malarial knowledge of private medicine retailers. They found that MoH interventions made considerable gains in medicine retailer knowledge and practice in the district of Makueni, made moderate gains in the district of Kwale, but made fewer gains in the district of Busia. Another intervention programme [12] made more improvements in reducing the sales of SP and increasing the rate of correct anti-malarial dosage in the district of Kisii than they did in the district of Kwale. The region in which these programmes were implemented was related to their success. Since intervention programmes of this kind are also costly, recommendations have been made to increase intervention efficacy by adapting them to the local context [14, 15], rather than implementing the same programme across the study area.

Geospatial analysis techniques can be used to meet this need by helping define the local context. These methods can find the distance between health facilities and residents [16], demonstrate how this distance affects treatment-seeking behaviour [17], and show whether behaviours are clustering in space [18]. These methods have been used to detect and predict clusters and spatio-temporal trends of malaria disease [19–22], to map malaria-related treatment and prevention behaviours [23–25], and have looked at the predictors to accessing effective malaria treatment within the retail sector [3, 35, 36]. Geospatial methods have also been used to inform interventions by showing where disease, and behaviours that relate to disease, are most prevalent [26]. However, geospatial techniques have infrequently been applied to improving rates of effective malaria treatment in the retail sector has been largely unexplored [23–25], and have not yet been applied with medicine retailers. With the proven success and continued recommendation of targeting interventions based on the results of spatial analyses [27, 28], and the multitude of intervention programmes designed to improve access to appropriate malaria treatment and implemented in the retail sector [6, 11–13, 29, 30], applying spatial analysis to medicine retailers’ anti-malarial behaviours presents an opportunity to support malaria eradication efforts.

The World Health Organization (WHO) has promulgated leveraging geospatial analysis techniques for malaria control and eradication. Senior advisors have published a call to use these techniques to support targeted interventions in malaria control, to lead to increases in cost-effectiveness, and intervention-effectiveness [26]. With the increase in attention medicine retailers are receiving as a result of programmes like AMFm, and the need to reduce costs by developing spatially- and behaviourally-targeted interventions, studies on the geospatial variation in medicine retailer behaviours are needed.

It is the objective of this research to identify determinants to medicine retailer recommendation and dispensation behaviours and to assess whether the strength of those associations varies across the study area. Understanding where each determinant is significantly related to medicine retailer behaviours will enable interventions to target these areas, advancing malaria treatment and control efforts in the region.

## Methods

### Study area and sample

**Fig. 1:**
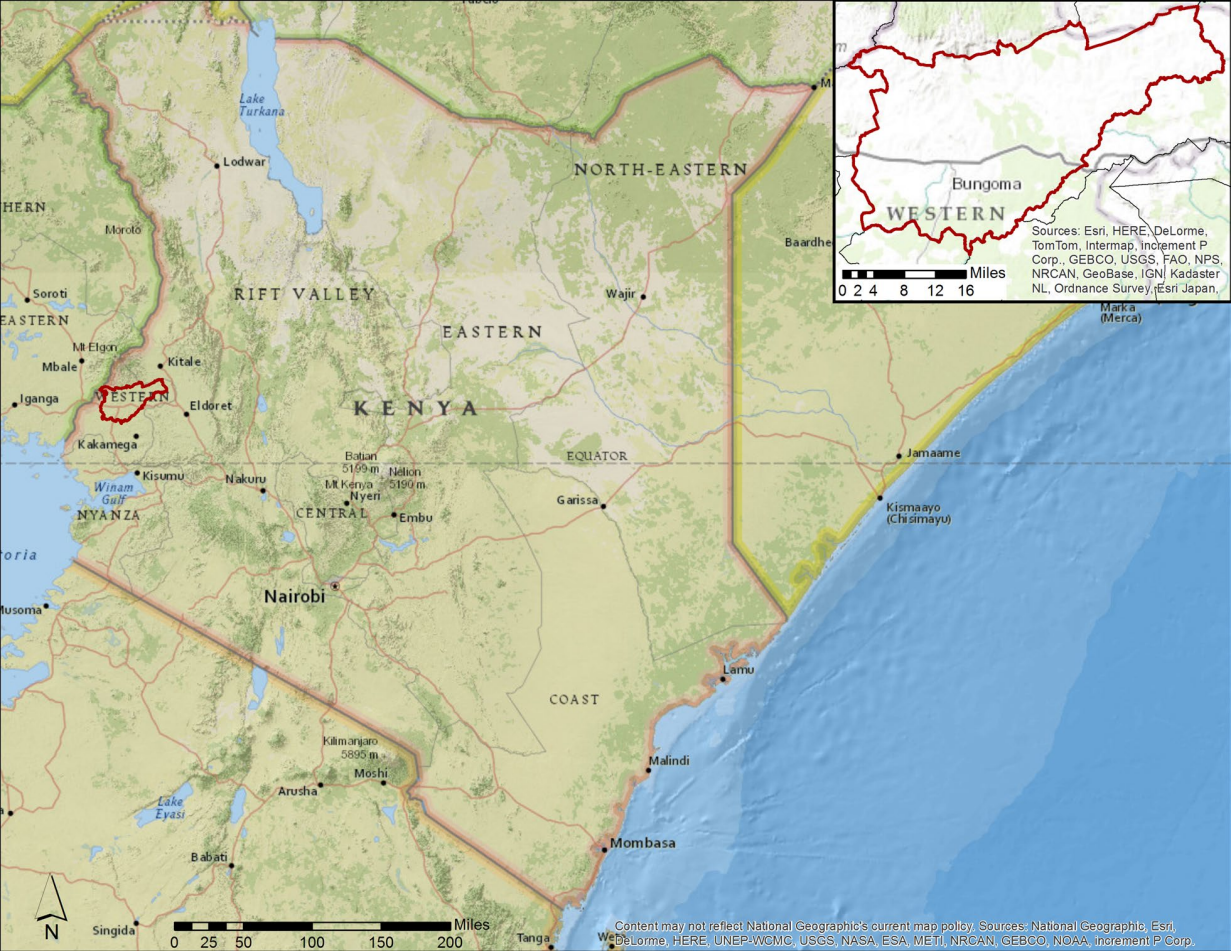
Map of Kenya, with the Bungoma district containing the study area inset

O’Meara and colleagues conducted a quantitative survey of private medicine retailers working in retail drug shops located within, or up to five kilometers from, the Webuye Health and Demographic Surveillance Site (WHDSS) [8, 16, 31]. The WHDSS is in the Bungoma district of western Kenya (Fig. 1). The study area is home to approximately 80,000 persons and has a primary economy of subsistence farming and that provided by a local sugar-processing factory [32]. The area is holo-endemic for malaria, with over 40% of a random sample of the people living in the Western Province having *Plasmodium falciparum* parasites in their peripheral blood [33].

### Data collection

Study staff were each assigned to cover a sub-section of the study area by motorbike. Staff visited every medicine retailer in their area in December of 2010, recording the name and GPS coordinates of each location. A quantitative survey tool was administered to one medicine retailer at each drug outlet location that met the inclusion criteria. Inclusion criteria were comprised of carrying anti-malarial medications and businesses that were privately owned. Exclusion criteria included locations that only sold general goods and sundries, were public health facilities, or refused participation. Further detail of data collection methods has been published elsewhere [16].

### Measures

The survey tool included eighty questions. Of the variables measured related to participant demographics, this analysis included respondent’s age, which was captured categorically and collapsed to the following categories: under 30, between 30 and 40, and over 40. Respondent’s education level was also included, which was captured in five categories (“none,” “some primary,” “completed primary,” “completed secondary,” and “some or competed above secondary”) and was collapsed into two categories, combining the upper two categories and the lower three categories. Respondent’s gender was also included in this analysis.

Factors related to health training included their type of health training, which was categorized as being untrained, having a training in pharmacy (including pharmacists, pharmacy technologists, and pharmacy assistants), or having a training in nursing/midwifery (including nurses, nurse aids, and midwives). Health qualification level was also included, which was categorized into no training, an assistant-level training, or a professional-level training. The binary variable of whether the respondent had ever attended a malaria workshop was also included. Variables related to characteristics of the drug shop included whether the shop offered diagnostic testing services, was open on weekends, was open more than 10 h a day or fewer than 10 h a day, and whether the shop had 1 staff member, 2 staff members, or 3-6 staff members.

The first outcome variable included in this analysis was whether a shop sold ACT medicines more than any other anti-malarial. This outcome was measured with the question, “Even if it is currently out of stock, which malaria medicine is most sold in your shop?” Responses were open-ended, and were coded either to ACT treatments, or to responses that included reference to any other anti-malarial therapy.

The second outcome analysed was which medication a medicine retailer would recommend to an adult with malaria. This outcome was measured with the question, “Which malaria medicine do you recommend most for adults.” Responses were coded in the same manner as the above question on anti-malarial drug knowledge, into “ACT” and “Not ACT” categories.

The third outcome analysed was which medication a retailer would recommend for a child under five with malaria. This outcome was measured with the question, “Which malaria medicine do you recommend most for children under 5 years of age.” Responses were coded in the same manner as the above two outcomes.

All of the above variables were selected for analysis based on a review of the literature, with weight given to the findings from studies conducted in the WHDSS [16, 31, 34–36].

### Data entry and analysis

All data collected from the survey were double entered into a Microsoft Access database and confirmed. Discrepancies were resolved by consulting the original survey forms. Variables from the survey that are of interest in this study were transposed to a binary format, coded and imported into Stata v11 [37]. All of the above variables were assessed using cluster analysis and found to have statistically significant spatial clusters. The results of this cluster analysis have been reported separately [18].

### Model building

Model building began with spatial cluster exploration of each variable considered for entry into the geographic spatial regression model. The results from this exploration have been reported separately [18]. Those variables that showed clustering in the exploratory spatial analysis were compared to each outcome using univariate logistic regression.

### Spatial analysis

Each of the statistically significant associations from the univariate analysis were included in pairwise comparisons of nested models. Those additions that improved goodness of fit measures were included in the final regression model for each variable. The global Moran’s *I* measure [38] was run on the residuals to detect spatial autocorrelation, using GeoDa v1.6 [39]. Geographic weighted regression (GWR) [40] was run on all three outcomes to investigate the potential for non-stationary relationships, or relationships that vary across space, between each outcome and the variables included in the final models, using GWR4 [41]. Geographically-weighted regression produces two models, a global regression model that assumes relationships are spatially stationary, occurring evenly across the study area, and a locally varying model that estimates local local-weighted regression for each point and its nearer neighbours. Resulting local parameter estimates are specific to each location. A GWR logistic model for binary data with an adaptive bi-square geographic kernel was used. The “golden section search” method was used to select the optimal bandwidth [41].

Comparisons of global and local models, and assessments of goodness of fit, were made using classic and corrected Akaike’s Information Criteria (AIC), according to its intended use with logistic regression models and the GWR4 software package [40, 42, 43], and deviance values [44]. The estimated regression coefficients from the local model were exported and mapped in ArcMap v.10.1 [45] to assess spatial variability in the coefficients. The pseudo R^2^ values for each model were also exported and mapped to assess local areas where the final models had the best fit. Finally, the residuals from the three GWR models were exported and analysed for spatial autocorrelation using Moran’s *I*, to ensure there was no residual spatial autocorrelation following the local geographic weighted regression.

### Ethics, consent, and permissions

The study that administered the survey received ethical review board approval from the Duke University Institutional Review Board in Durham, North Carolina, and the Moi University Institutional Research and Ethics Committee in Eldoret, Kenya. The study conducting the analysis received approval from the University of Texas Health Science Center at Houston Committee for the Protection of Human Subjects, and was assigned IRB protocol number HSC-SPH-14-1035.

The researchers conducting the original study, from which survey data was analyzed in this study, held meetings with key members of the community, including village elders, community chiefs, and assistant chiefs to discuss the objectives of the survey and garner their approval. Key leaders shared survey objectives with their communities. Each participant in the survey gave verbal consent prior to the start of the survey.

## Results

**Fig. 2:**
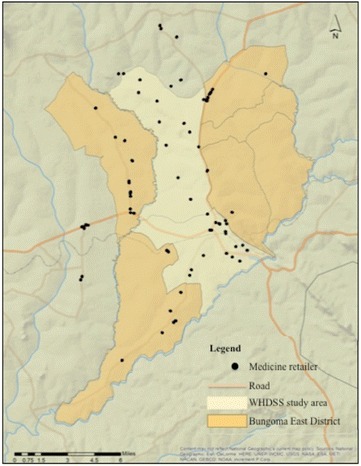
Map of the WHDSS study area showing shop locations, adapted from Rusk et al. [18]

The original mapping exercise identified 117 retailers located within or accessible to the WHDSS. Surveys were completed from ninety-seven unique shop locations. Of these, six either did not include GIS coordinates of the shop location (n = 4), or recorded the coordinates incorrectly (n = 2), and were excluded from this analysis. A map of the study area, showing shop locations, is available in Fig. 2. Because GWR4 cannot run analyses on missing data, those surveys that had missing observations for any of the variables included in the analyses were dropped (n = 4). The final analysis included 74% of the retailers located within or accessible to the WHDSS. Of the eighty-seven surveys that were included, 77% were completed by females, 44% were between the ages of 30 and 40 years, 68% had above a secondary school education, and 86% had health-related training. The 10 observations excluded from the analysis had no significant differences from those included, and had similar demographics. Of those excluded, 58% were female, 50% were between 30 and 40 years old, 75% had above a secondary education, and 83% had health-related training.

### Results from univariate logistic regression

Each variable that showed statistically significant clustering in the spatial cluster analysis was compared to each outcome using univariate logistic regression. Those comparisons were considered significant, and so eligible for entry into the multivariate geographic weighted regression model, at the *p* < 0.2 level. The results of the univariate regression are listed below, separated by outcome.

### Recommending correct treatment to adults

Of the medicine retailer demographic variables, only two were found to have a statistically significant relationship with the outcome of recommending ACT to treat malaria in adults. These two include the level of health qualification and the type of health qualification. The associations of all demographic variables with recommending correct malaria treatment to adults are described in Table 1.

**Table 1:** Association between medicine retailer demographics and study outcomes

	% (n)	Unadjusted odds ratios and (95% confidence intervals; p values)		
		Recommended ACTs as malaria treatment for adult patients	Recommended ACTs as malaria treatment for under 5 patients	Reported selling ACTs more than any other antimalarial
Age				
Under 30	38% (33)	1 (reference)	1 (reference)	1 (reference)
Between 30 and 40	44% (38)	0.65 (0.25–1.67; 0.371)	1.08 (0.42–2.75; 0.872)	0.99 (0.38–2.54; 0.978)
Over 40	17% (15)	0.98 (0.28–3.39; 0.968)	0.80 (0.23–2.76; 0.724)	0.90 (0.26–3.13; 0.875)
Gender				
Female	77% (67)	1 (reference)	1 (reference)	1 (reference)
Male	23% (20)	1.7 (0.60–4.79; 0.317)	0.59 (0.21–1.66; 0.317)	0.93 (0.34–2.57; 0.887)
Education level				
Secondary or below	32% (28)	1 (reference)	1 (reference)	1 (reference)
Above secondary	68% (59)	1.1 (0.45–2.71; 0.836)	1.4 (0.56–3.48; 0.475)	1.42 (0.56–3.59; 0.461)
Health qualification type				
Untrained	14% (12)	1 (reference)	1 (reference)	1 (reference)
Pharmacy	40% (35)	5.75 (1.31–25.29; 0.021)**	1.5 (0.38–5.93; 0.563)	5.29 (1.01–27.75; 0.049)**
Nurse/midwife	46% (40)	3.67 (0.86–15.59; 0.079)*	2 (0.52–7.72; 0.315)	3.33 (0.64–17.26; 0.151)*
Health qualification level				
Untrained	14% (12)	1 (reference)	1 (reference)	1 (reference)
Assistant level	25% (22)	3.0 (0.67–14.15; 0.165)*	2.4 (0.55–10.38; 0.241)*	5 (0.88–28.29; 0.07)*
Professional level	61% (53)	5.37 (1.29–22.26; 0.021)**	1.53 (0.41–5.73; 0.525)	3.83 (0.76–19.22; 0.102)*
Attended malaria workshop				
No	59% (51)	1 (reference)	1 (reference)	1 (reference)
Yes	41% (35)	1.63 (0.68–3.92; 0.277)	1.64 (0.69–3.91; 0.264)	2.38 (0.98–5.75; 0.055)*

* Significant at p < 0.2

** Significant at p < 0.05

The odds of recommending correct treatment to adults increased with the level of health qualification. Assistants were three times more likely to recommend ACT to treat malaria in adults than those without health-related training (OR = 3.0, 95% CI 0.67–14.15; *p* = 0.165), while professionals were five times more likely to recommend appropriate treatment (OR = 5.37, 95% CI 1.29–22.26; *p* = 0.021). Health-qualification type was also associated with recommending the correct anti-malarial to adults. Those trained in pharmacy were five times more likely to recommend ACT to adults (OR = 5.75, 95% CI 1.31–25.29; *p* = 0.021) compared to those without health-related training. Those trained in nursing or midwifery were also more likely to recommend correct treatment (OR = 3.67, 95% CI 0.86–15.59; *p* = 0.079) than those without training.

Table 2 depicts the relationships between medicine retailer knowledge and behaviours with the outcome of recommending ACT to treat malaria in adults. Correctly identifying ACT as the MoH-recommended first-line treatment for malaria increased the likelihood of recommending that treatment to adults compared to those who did not correctly identify the recommended malaria therapy (OR = 3.0, 95% CI 1.21–7.47; *p* = 0.018). Selling ACT medicines more than any other anti-malarial also increased the likelihood of recommending it to adults compared to retailers who did not sell ACT medicines more than other anti-malarials (OR = 8.89, 95% CI 3.11–25.39; *p* < 0.001). Recommending ACT to children was also statistically significantly associated with recommending it to adults (OR = 5.37, 95% CI 2.08–13.87; *p* = 0.001) compared to retailers who did not recommend ACT to children.

**Table 2:** Association between medicine retailer knowledge, behaviours and study outcomes

	% (n)	Unadjusted odds ratios and (95% confidence intervals; p-values)		
		Recommended ACTs as malaria treatment for adult patients	Recommended ACTs as malaria treatment for under 5 patients	Reported selling ACTs more than any other antimalarial
Identified ACT as MOH-recommended antimalarial				
No	37% (32)	1 (reference)	1 (reference)	1 (reference)
Yes	63% (55)	3.0 (1.21–7.47; 0.018)**	2.34 (0.93–5.88; 0.070)*	2.36 (0.92–6.03; 0.073)*
Recommended ACT to adults				
No	45% (39)	–	1 (reference)	1 (reference)
Yes	55% (48)	–	5.37 (2.08–13.87; 0.001)**	8.89 (3.11–25.39; < 0.001)**
Recommended ACT to children				
No	55% (48)	1 (reference)	–	1 (reference)
Yes	45% (39)	5.37 (2.08–13.87; 0.001)**	–	3.76 (1.52–9.28; 0.004)**
Sold ACTs more than any other antimalarial				
No	59% (51)	1 (reference)	1 (reference)	–
Yes	41% (36)	8.89 (3.11–25.39; < 0.001)**	3.76 (1.52–9.28; 0.004)**	–
Gave Fansidar to a mother requesting it				
No	81% (70)	1 (reference)	1 (reference)	1 (reference)
Yes	18% (16)	0.67 (0.21–2.01; 0.461)	0.36 (0.11–1.25; 0.109)*	0.94 (0.30–2.94; 0.919)

* Significant at p < 0.2

** Significant at p < 0.05

All of the retail drug shop characteristic variables had statistically significant relationships with recommending ACT to adults. Shops that offered diagnostic testing increased the likelihood three-fold that the retailer working in that shop recommended ACT to adults (OR = 3.16, 95% CI 0.62–16.17; *p* = 0.167), compared to shops that did not offer testing. These odds also increased with each additional staff member working in the shop. Shops with two staff members were almost two times more likely to recommend ACT than shops with one staff member (OR = 1.94, 95% CI 0.76–5.0; p = 0.168). Shops with between three and six staff members were five times more likely to recommend ACT than shops with one staff member (OR = 5.4, 95% CI 0.59–49.47; p = 0.136). All shop level characteristics and their association with recommending correct treatment to adults can be found in Table 3.

**Table 3:** Association of retail drug shop characteristics and study outcomes

	% (n)	Unadjusted odds ratios and (95% confidence intervals; p values)		
		Recommended ACTs as malaria treatment for adult patients	Recommended ACTs as malaria treatment for under 5 patients	Reported selling ACTs more than any other antimalarial
Offers diagnostic testing				
No	90% (78)	1 (reference)	1 (reference)	1 (reference)
Yes	10% (9)	3.16 (0.62–16.17; 0.167)*	0.58 (0.14–2.5; 0.468)	1.15 (0.29–4.62; 0.844)
Total number of staff				
Only 1 staff member	60% (52)	1 (reference)	1 (reference)	1 (reference)
2 staff members	32% (28)	1.94 (0.76–5; 0.168)*	1.48 (0.59–3.72; 0.409)	1.74 (0.68–4.41; 0.245)*
3–6 staff members	7% (6)	5.4 (0.59–49.47; 0.136)*	2.95 (0.5–17.6; 0.235)*	1.74 (0.32–9.48; 0.524)
Open weekends				
Close weekends	17% (15)	1 (reference)	1 (reference)	1 (reference)
Open weekends	83% (72)	2.1 (0.68–6.53; 0.2)*	1.27 (0.41–3.94; 0.68)	2.2 (0.64–7.57; 0.211)*
Open hours per day				
Fewer than 10 h	60% (52)	1 (reference)	1 (reference)	1 (reference)
10 or more hours	39% (34)	1.83 (0.75–4.46; 0.181)*	0.51 (0.21–1.23; 0.132)*	1.74 (0.72–4.18; 0.218)*

* Significant at p < 0.2

** Significant at p < 0.05

### Recommending correct treatment to children

The only medicine retailer demographic variable that had a statistically significant relationship with recommending ACT to treat malaria in children was having a health-related qualification at the assistant level (OR = 2.4, 95% CI 0.55–10.38; *p* = 0.241) when compared to untrained staff, but not at the professional level. However, all of the retailer knowledge and behaviour variables had statistically significant relationships with recommending the correct anti-malarial to children (Table 2). Retailers who identified the MoH-recommended anti-malarial drug, compared to those who did not, were twice as likely to recommend it to children (OR = 2.34, 95% CI 0.93–5.88; *p* = 0.070), and those who recommended ACTs to adults were five times as likely to recommend it to children (OR = 5.37, 95% CI 2.08–13.87; *p* = 0.001) compared to those who did not recommend ACT to adults. Selling ACT medicines more than any other anti-malarial also increased the odds of recommending it to children (OR = 3.76, 95% CI 1.52–9.28; *p* = 0.004). Contrastingly, retailers who would sell Fansidar**®** to a mother requesting the drug by name for her three year old child she believes has malaria were less likely to recommend an ACT to treat malaria in a child (OR = 0.36, 95% CI 0.11–1.25; *p* = 0.109) compared to retailers who would not sell Fansidar^**®**^.

Like recommending correct treatment to adults, the total number of staff working in a shop was also related to recommending correct treatment to children (Table 3). Shops with between 3 and 6 staff members were more likely to recommend ACT than shops with only one staff member (OR = 2.95, 95% CI 0.5–17.6; *p* = 0.235). However, retailers working in shops with longer open hours were less likely to recommend appropriate anti-malarials to children than shops open fewer than ten hours a day (OR = 0.51, 95% CI 0.21–1.23; *p* = 0.132).

### Selling ACT medicines more than any other anti-malarial

Health-qualification type and level were both related to medicine retailers selling ACT medicines more than any other anti-malarial (Table 1). Having training in pharmacy or having an assistant level training increased this likelihood five times (OR = 5.29, 95% CI 1.01–27.75; *p* = 0.049) and (OR = 5.0, 95% CI 0.88–28.29; *p* = 0.07), respectively, when compared to untrained staff. Similarly, having training in nursing/midwifery or at the professional level increased this likelihood by three times (OR = 3.33, 95% CI 0.64–17.26; *p* = 0.151) and (OR = 3.83, 95% CI 0.76–19.22; *p* = 0.102), respectively, when compared to untrained staff.

Recommending ACT to adults and to children were both related to selling ACT medicines more than any other anti-malarial (Table 2). However, retailers who identified ACT as the MoH-recommended treatment for malaria were also more likely to sell ACT medicines more than other anti-malarial drugs (OR = 2.36, 95% CI 0.92–6.03; *p* = 0.073) compared to retailers who did not correctly identify the MoH-recommended anti-malarial. Retailers who worked in shops that were open weekends, had two staff members, or were open more than 10 h per day were also more likely to sell more ACT medicines, but these relationships were not included in the spatial analysis because they did not meet the statistical significance levels set a priori (Table 3).

### Results from spatial analysis

The determinants included after pairwise comparison for the outcome of recommending the correct treatment to adults were recommending that treatment to children, selling that treatment more than other anti-malarial therapies, identifying that treatment as the MoH first-line recommendation, and having health-related training. Determinants included in the final model for recommending ACT to children include recommending it to adults, selling it more than other anti-malarials, identifying it as the MoH recommending recommended treatment, giving Fansidar® to a mother requesting it, offering diagnostic testing, and gender. Though gender was not found to be statistically significantly associated with treating children appropriately, it was retained in the final model due to evidence in past research that indicate female retailers are more likely to recommend and dispense appropriate treatment to children than male retailers [8]. For the outcome of selling ACT medicines more than other therapies, the determinants included in the final model were recommending to adults, to children, identifying ACT as the first-line anti-malarial drug and having attended a malaria workshop.

Multivariate logistic regression was run on each final model, and the residuals were predicted from that model for each outcome. The global Moran’s *I* measure was used to detect the presence of spatial autocorrelation in each model. The residuals from the final logistic model for the outcome of recommending the correct treatment to adult patients revealed spatial autocorrelation (Moran’s *I* = 0.10, z = 1.66, pseudo *p* = 0.048). For the outcome of recommending the correct treatment to children, the Moran’s *I* did not reject the null hypothesis of no spatial autocorrelation (Moran’s *I* = 0.05, z = 0.88, pseudo *p* = 0.178). The residuals from the final model for selling ACT medicines more than any other anti-malarial also had spatial autocorrelation (Moran’s *I* = 0.13, z = 2.06, pseudo *p* = 0.027).

Geographic weighted regression was run on all three models to investigate whether relationships between the outcome behaviour and the determinants varied across space. The local parameter estimates for each model are summarized in Tables 4, 5 and 6. In these tables, statistical significance indicates that those variables vary locally, within the study area. Those variables that are not statistically significant are not varying locally, but rather are fixed globally across the study area.

**Table 4:** Parameter estimates from the geographically geographically-weighted global regression model for the outcome of recommending the correct malaria treatment to adults

	Estimate	Standard error
Intercept	−2.331	0.88*
Identified ACT as MOH-recommended antimalarial	0.645	0.559
Recommended correct treatment to children under 5	1.307	0.544*
Had health-related training	1.052	0.865
Sold ACTs more than any other antimalarial	1.787	0.566*

* Denotes statistical significance

**Table 5:** Parameter estimates from the geographically geographically-weighted global regression model for the outcome of recommending the correct malaria treatment to children under five

	Estimate	Standard error
Intercept	−1.354	0.522*
Identified ACT as MOH-recommended antimalarial	0.602	0.54
Recommended correct treatment to adults	1.411	0.562*
Gave Fansidar to a mother requesting it	−0.982	0.685
Sold ACTs more than any other antimalarial	0.825	0.545
Female	−0.89	0.621

* Statistical significance

**Table 6:** Parameter estimates from the geographically geographically-weighted global regression model for the outcome of selling ACT medicines more than any other anti-malarial

	Estimate	Standard error
Intercept	−2.407	0.615*
Identified ACT as MOH-recommended antimalarial	0.448	0.561
Recommended correct treatment to children under 5	0.742	0.533
Recommended correct treatment to adults	1.8	0.58*
Attended malaria workshop	0.781	0.521

* Statistical significance

In the outcome of recommending ACTs to treat malaria in adults, the factors selling ACTs more than other anti-malarials and recommending ACTs to children have local variation, indicating that the relationship between these variables and the outcome is spatial (Table 4). In the outcome of recommending ACTs to children, the factors recommending that treatment to adults is the only variable with local variation (Table 5). For the outcome of selling ACTs more than any other anti-malarial, only the factor recommending that treatment to adults had local variation (Table 6).

All variables included in the global model were also tested in the local model. Comparison of AIC values and deviance values for each outcome are summarized in Table 7. These comparisons indicate that the local model was a better fit than the global model for both the recommending correct treatment to adults outcome and the recommending correct treatment to children outcome. Using the standard expected decrease of the AIC by 3 points to constitute better fit [40], the outcome of selling ACT medicines more than any other anti-malarial model was not a better fit with the local model, indicating that the relationships in this model were spatially stationary across the study area and a global spatial model is appropriate. However, both the recommends correct treatment to adults and recommends correct treatment to children outcome models have lower AIC, AICc, and deviance values, indicating that the local model is a better fit for the data, and that spatial heterogeneity exists in the data.

**Table 7:** Comparison of global and local Logistic model diagnostics for each outcome

	Global model	Local model
Recommended correct treatment to adults		
Akaike information criterion	97.85	91.47
Corrected akaike information criterion	98.58	92.47
Deviance	87.85	79.78
Recommended correct treatment to children under 5		
Akaike information criterion	106.95	99.07
Corrected akaike information criterion	108.01	101.33
Deviance	94.95	81.49
Sold ACTs more than any other antimalarial		
Akaike information criterion	101.28	100.75
Corrected akaike information criterion	102.03	102.04
Deviance	91.28	87.48

**Fig. 3:**
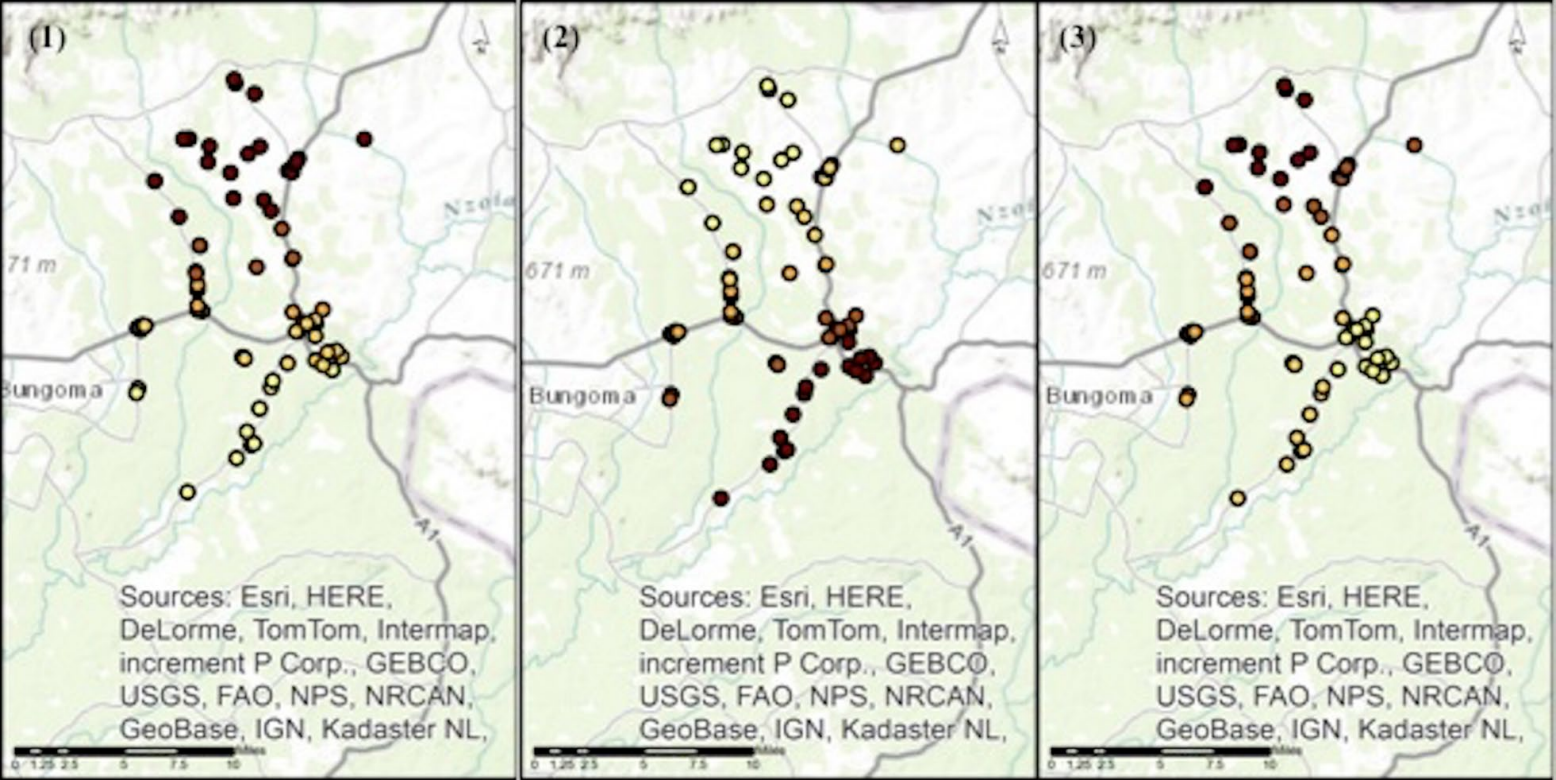
Local coefficient estimates for the statistically significant determinants to the outcome recommending correct antimalarial treatment to adults, across the Webuye Demographic Health Surveillance Site study area. *Panel 1* shows the local coefficient estimates for the statistically significant determinant recommending the correct antimalarial treatment to children under 5 *Panel 2* shows the local coefficient estimates for the statistically significant determinant having health-related training *Panel 3* shows the local coefficient estimates for the statistically significant determinant correctly identifying ACTs as the MOH-recommended first-line treatment for uncomplicated malaria The darker the* circle* indicates the stronger the association

**Fig. 4:**
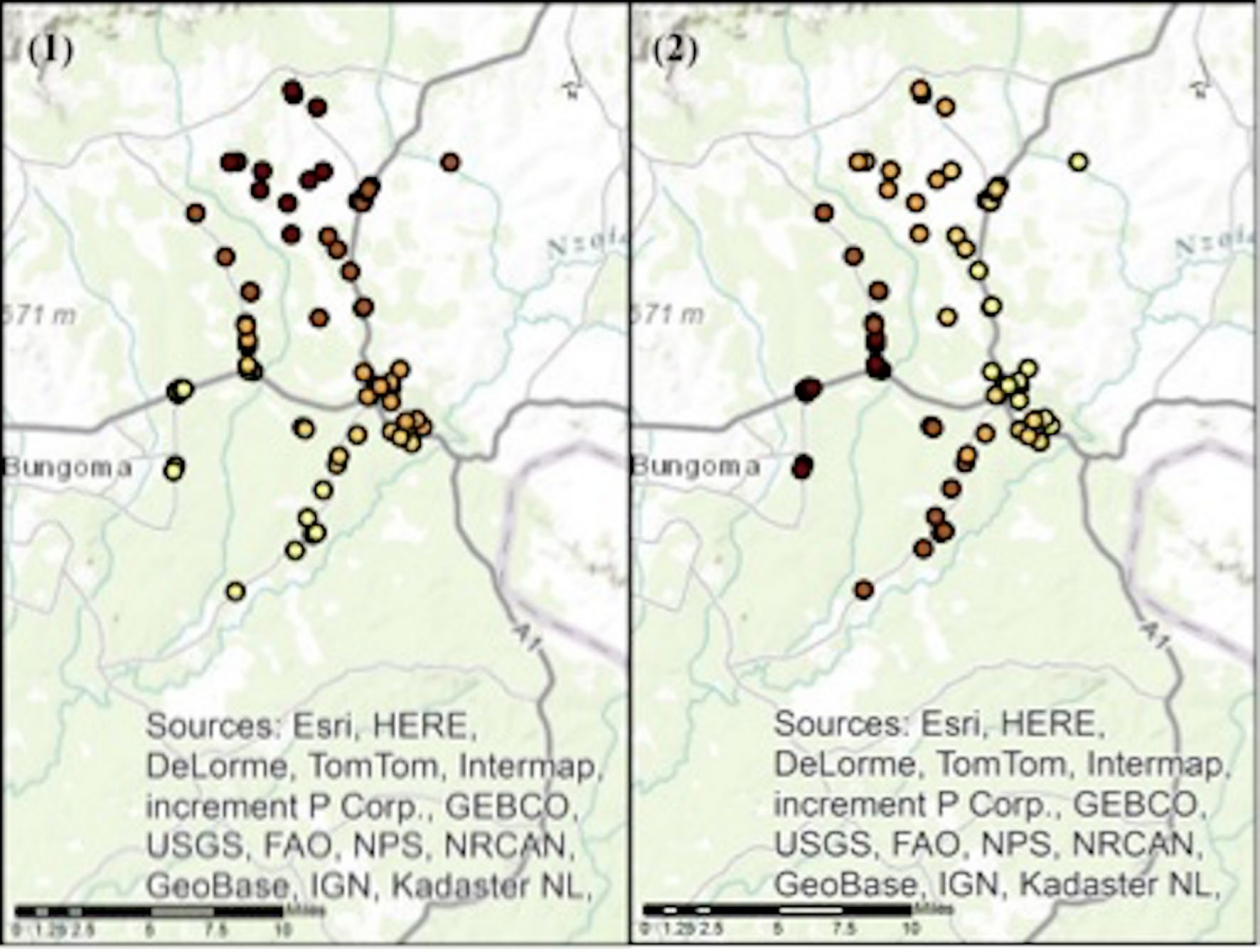
Local coefficient estimates for the statistically significant determinants to the outcome recommending correct antimalarial treatment to children under 5, across the Webuye Demographic Health Surveillance Site study area. *Panel 1* shows the local coefficient estimates for the statistically significant determinant recommending correct treatment to adults *Panel 2* shows the local coefficient estimates for the statistically significant determinant giving Fansidar® to a mother requesting it The darker the* circle* indicates the stronger the association

**Fig. 5:**
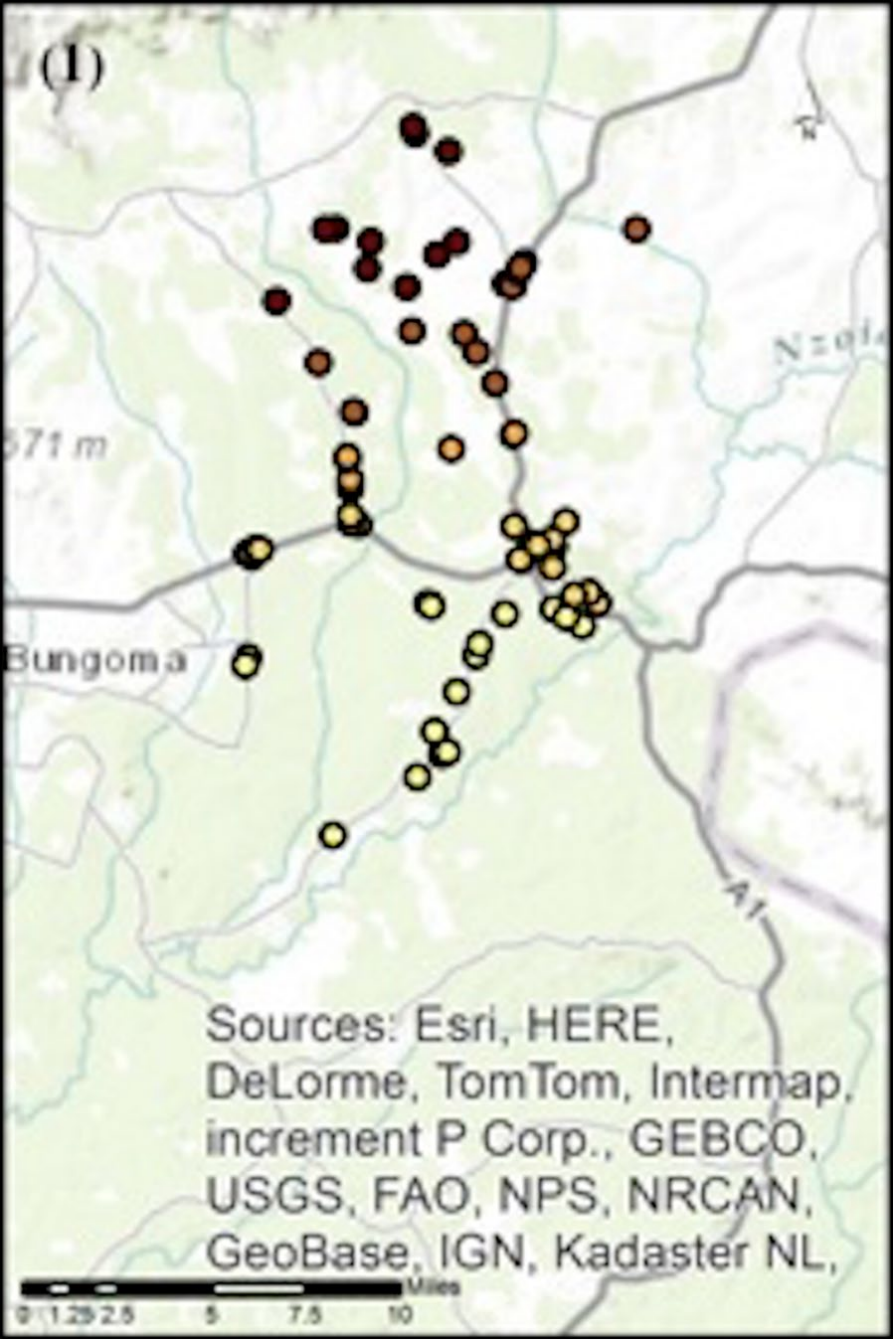
Local coefficient estimates for the statistically significant determinant to the outcome selling ACTs more than any other antimalarial medication, across the Webuye Demographic Health Surveillance Site study area. *Panel 1* shows the local coefficient estimates for the only statistically significant determinant: having attended a malaria workshop The darker the* circle* indicates the stronger the association

Model performance was also explored visually in the data by mapping the local coefficient estimates of the significant predictor variables from the local models of each outcome, with the darker the circle indicating the stronger the association (Figs. 3, 4 and 5).

These visualizations reveal that the association between having training in a health-related field and recommending the correct anti-malarial treatment to adults is strongest in the peri-urban centre of Webuye Town and those locations to the southwest of that centre. In contrast, identifying the correct MoH-recommended anti-malarial treatment, and recommending that treatment to children, have weak associations near the peri-urban centre, with the strongest associations to the north of the study area.

The association between having attended a malaria workshop and selling ACT medicines more than any other anti-malarial is also strongest in the north of the study area. The association between giving Fansidar^®^ to a mother who requests it and recommending the correct anti-malarial treatment to children has the strongest associations due west of the peri-urban centre, in the southwest of the study area.

**Fig. 6:**
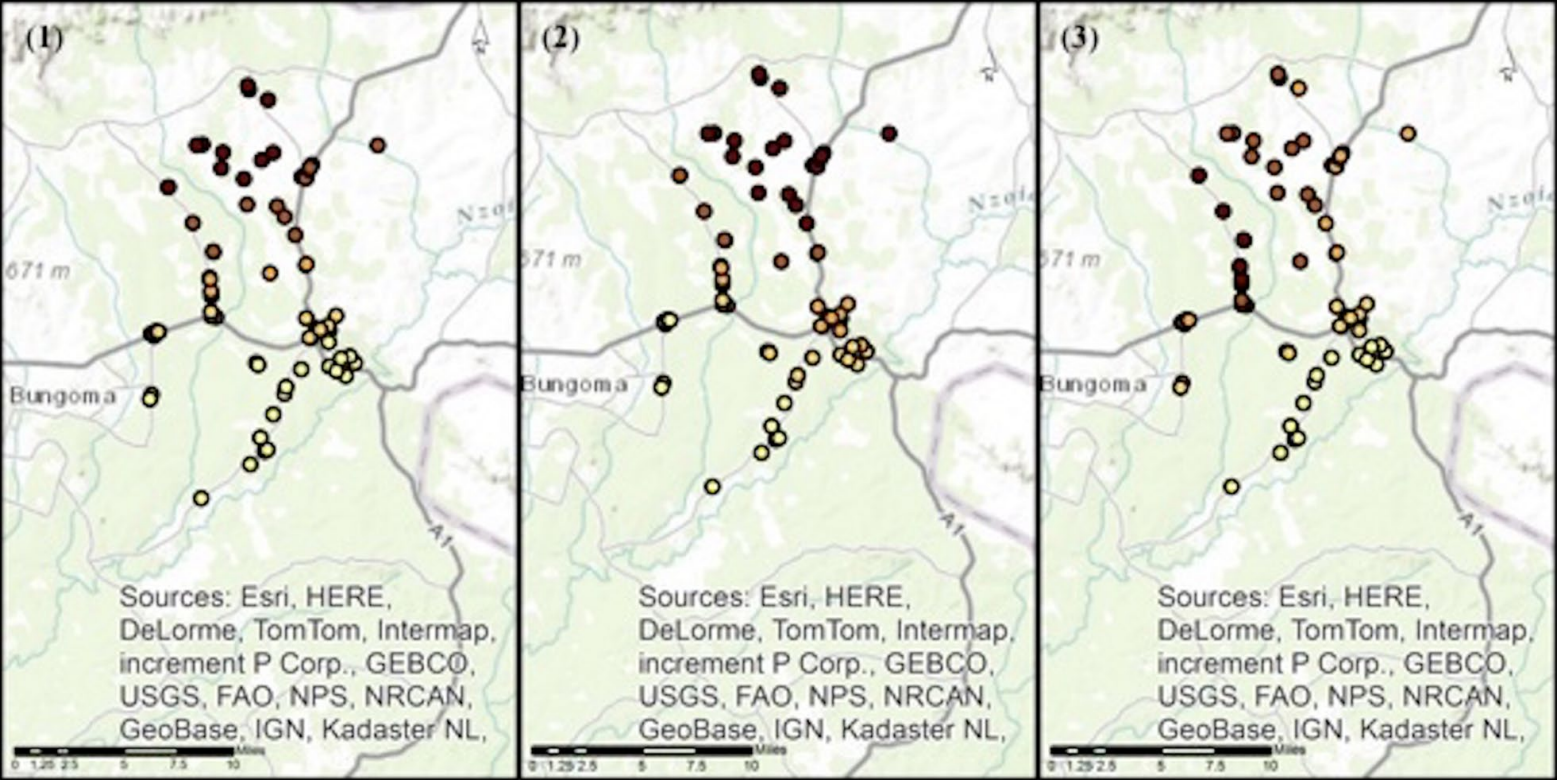
Spatial distribution of pseudo-R^2^ values for the full model of each outcome, across the Webuye Demographic Health Surveillance Site study area. *Panel 1* shows the spatial distribution of pseudo-R^2^ values for the full model of the outcome recommending correct treatment to adults *Panel 2* shows the spatial distribution of pseudo-R^2^ values for the full model of the outcome recommending correct treatment to children under 5 *Panel 3* shows the spatial distribution of pseudo-R^2^ values for the full model of the outcome selling ACTs more than another antimalarial The darker the* circle* indicates the better the model fit

The spatial distribution of the pseudo-R^2^ values across the study area indicates that the included variables (shown in Tables 1, 2 and 3) have stronger associations with the outcome where the darkest dots are seen, compared to elsewhere in the study area. The models for the outcomes of recommending correct treatment to adults and to children have the best model fit in the northern part of the study area (see parts 1 and 2 of Fig. 6). Comparatively, the outcome of selling ACT medicines more than any other anti-malarial has the best model fit to the west of the peri-urban centre (see part 3 of Fig. 6).

The residuals of the geographic weighted regression model were tested for spatial autocorrelation using Global Moran’s *I*. All outcome models yielded non-significant Moran’s *I*: recommending correct treatment for adults (Moran’s *I* = −0.09, z = −1.11, pseudo *p* = 0.13), for children (Moran’s *I* = −0.1, z = −1.34, pseudo *p* = 0.08), and selling ACT medicines more than any other anti-malarial outcome: Moran’s *I* = 0.02, z = 0.37, pseudo *p* = 0.33. These results indicate that the geographic weighted logistic regression was successful in addressing the spatial autocorrelation existent in the data.

## Discussion

These analyses detected two spatial areas of interest to malaria control efforts. The first, to the west of the study region in an area called Bokoli, showed a spatial relationship between a mother demanding Fansidar^®^ for her child and a provider recommending appropriate treatment to a child with malaria. This relationship coincides in space with a previous finding that a greater number of nursing-trained providers, compared to pharmacy-trained providers, worked in this area over what was expected [18]. It could be that, in this area, far from the peri-urban centre, the district hospital, and the concentration of pharmacy-trained staff working in shops, nursing-trained staff are more likely to yield to a mother’s demands when dispensing anti-malarial medication. Additional research is required to discover what is unique to the Bokoli area that resulted in these findings.

The second area of interest was in the north of the study area, in an area known as Misikhu, where the relationships between attending a malaria workshop was spatially correlated with selling ACT more than another anti-malarial, and between knowing the MOH-recommended anti-malarial was spatially correlated with recommending that drug to adults. This not only reveals notable spatial heterogeneity in these relationships, indicating that geography is important when understanding medicine retailer behaviours, but also a spatial phenomenon that is occurring in the north of the study area. This study concurs with previous studies that found inconsistencies in medicine retailer behaviour across administrative boundaries [6, 12] in that the shops in this part of the study region lie outside the boundaries of the WHDSS. Given that multiple behaviours are converging in this area, it is a prime target for interventions focused on improving medicine retailer behaviours for malaria treatment and control. These findings are also important when considering the impact political boundaries have on health outcomes and behaviours, as they often dictate where funds are spent and where intervention efforts are focused.

These analyses demonstrate that traditional aspatial regression analyses do not completely capture the relationship between retail provider characteristics and their drug dispensing behaviours. This study also reveals that spatial correlation between malaria case management behaviours and provider knowledge, training, and recommending behaviours are behaviour-dependent as well as space-dependent. In other words, a one-size-fits all approach to intervening with the hopes of changing malaria case management behaviours in this study area would likely be less successful than a customized, tailored approach that would address the specific behaviours and outcomes that are most related in that specific sub-region. These findings provide support to the recommendations of Carter et al. [26], that national malaria control programmes begin to incorporate spatial targeting of their interventions.

From the multivariate geographic weighted regression, statistically significant relationships were found between selling ACT medicines more than other anti-malarials, and recommending ACT to adults. Unexpectedly, statistically significant spatial relationships were not found between provider training or education and recommendation behaviour, indicating that interventions in this area should focus on the provider-customer relationship, over the traditional provider education interventions.

Statistically significant spatial relationships were also found between recommending ACT to children under five and recommending ACT to adults. However, significant spatial relationships were not found between recommendation behaviour to children and provider gender, as had been previously postulated [8]. Past studies on this population have found significant relationships between provider behaviour and gender, but when considered in the light of the spatial analyses, it can be suggested that these relationships are not spatial. Rather, the relationships between recommendation behaviours are spatial. Interventions hoping to improve provider recommendation, and with it, dispensation behaviour, should consider that recommendation behaviour is spatially associated (both with adults and with children). A potential explanation for this is that these behaviours are learned from retailers working in proximity to one another, where the relationship between gender and behaviour are not due to proximity. This discovery supports the train-the-trainer approaches in interventions. This finding supports those by Tavrow and colleagues [13], who evaluated a train-the-trainer programme among medicine retailers in the same district of Western Kenya. They found that training mobile vendors and wholesale counter attendants, who then in turn trained providers in retail outlets, resulted in improvements in malaria drug knowledge, stocking patterns, and prescribing practices in retail drug shops. This intervention was also found to be cost effective given its investment and impact. Leveraging geospatial analyses of provider behaviours can provide targets for similar interventions.

As the data in this study were collected from self-reports, and were not directly observed by study staff, they are vulnerable to reporting bias. Interviewers did not request copies of training credentials or professional licenses to verify participant responses on health training, qualification, or workshop attendance. However, self-report is more likely to favor a higher or better training than a lesser, indicating that the true effect on study outcomes may be understated. The outcome of ACT sales was also self-reported and may be vulnerable to recall bias, as shop sales data were not analysed in this study. This outcome is also dependent on anti-malarial stocks, and as such, sensitive to drug stockouts. However, previous data analysis determine that ACT (in this case, AL), were not more likely to have been out of stock than other anti-malarials including SP, amodiaquine, or quinine, within the last month or on the day of the survey [16]. Generalizability of study findings may be limited due to the restricted geographic area studied. However, for those geographic areas that have a similar malaria endemicity profile, with a shared reliance on the retail sector for malaria case management, these findings could be meaningful, particularly as it relates to the application of geospatial analyses to understanding retailer behaviour.

## Conclusion

The results of this study demonstrate how spatial analysis can provide valuable information for interventions, revealing where interventions are most needed, and where the relationship between determinants and target behaviours are strongest. This information tells interventionists not only where to focus efforts, but it supports the prioritizing of intervention activities by concentrating on those behaviours most strongly associated with the outcome of interest. To effectively address low rates of appropriate malaria case management beliefs and behaviours, policymakers are advised to consider the context-specific determinants to these behaviours. These findings are also useful when choosing where and how to spend malaria control funds, in that it highlights the need for intervention in specific spatial areas.

Spatial analyses have been incorporated into national malaria control programmes to predict malaria risk and examine the distribution of malarial incidence across tie and space. These activities have supported the effective targeting of malaria infection clusters. However, the role of spatial analysis in malaria eradication has not yet been expanded to the spatial distribution of behaviours related to malaria control. Given the interest in effectively managing budgets allocated to malaria intervention, the role of behaviour in effective malaria control, and the necessity of supporting treatment and control efforts as efficiently as possible, geospatial analysis of behavioural determinants offers a promising advancement toward eradication efforts.
